# Some Remarks on Dr. Bent's Cases of Varioloid Disease after Vaccination

**Published:** 1819-04

**Authors:** Richard Pew

**Affiliations:** Sherborne, Dorset, Member of the Royal Medical and other Societies of Edinburgh.


*For the London Medical and Physical Journal.*

### *Some Remarks on Dr. Bent's Cases of Varioloid Disease after Vaccination;* by Richard Pew, M.D. of Sherborne, Dorset, Member of the Royal Medical and other Societies of Edinburgh.

THE long-agitated question, Whether vaccination be or be not a *sufficient* safeguard against all danger from small-pox infection? and, consequently, Whether vaccination should be preferred to variolation? are questions of such vital importance to the comfort and happiness of parents and guardians, and to the well-being of society at large, that every thing offered to the public respecting them ought to be as clear and as distinct as possible. I was led into these reflections by the paintings of two cases in your valuable Journal, for December 1818, of an eruptive disease, which Dr. Bent, of Derby, *seems*, from the title of his paper, to set down as cases of *varioloid** disease *after vaccination*; although Joseph Pegg, from whom the first painting was taken, "said that he had been inoculated for cow-pox *without effect*," and seems (although we are not informed of the fact) never to have had the small-pox: whilst, in respect to the subject of the second picture, marked A. B., we are left in perfect ignorance whether the patient had ever been attempted to be vaccinated or variolated at all!

I do not mean to insinuate that Dr. Bent intended to deceive or to steal a march upon the public; on the contrary, it seems to be his wish to be strictly impartial: but the manner and the order in which his cases are related seem calculated to lead to confusion. I shall first endeavour, therefore, to put these cases into what appears to me a more distinct order, and then to make a few observations upon them. For the sake of brevity, I shall omit dates and other minutiæ, and attend to those essential points only in which, in my opinion, the gest of the arguments will turn.

Mary Miller, then, was taken into the hospital at Derby, with an eruptive disease upon her: she had, a few days before, been delivered of a child. This person had, it seems, "been at a house where two children were *supposed* to have just had the small-pox, one of whom died." (Unfortunately, no enquiry seems to have been made into the real nature of the disease of these children, nor from what source it was derived.)

Two children, who had taken infection from the bed on which Mary Miller was delivered, had an eruptive disease; which three or four of Dr. Bent's medical friends did *not hesitate* in *pronouncing* to be *chicken-pox!*

Could three or four medical men, with the aid, as we must suppose, of the Doctor himself, be mistaken in their unanimous opinion of its being chicken-pox?—the thing seems next to impossible; and, if they were *not mistaken*, could the disease of Mary Miller have been *small*-pox?—this also is impossible; and, if the disease of Mary Miller was *not small-pox*, could the disease of the children, from whom Mary Miller took the infection, have been small-pox?—this seems also to be impossible. Can we gather grapes off thorns, or figs off thistles? And, if the case of Mary Miller, and of the children from whom she took her disease, were

---

* This title was not applied to the paper in question by Dr. Bent: we used it as a convenient, if not a strictly appropriate, appellation.—Edit.

*not* cases of small-pox, could the diseases of Joseph Pegg, of the young gentleman who was a pupil of the hospital, or, in short, any other of the persons who took it from Mary Miller, have been cases of small-pox? Could Mary Miller communicate to others a disease she was not affected with herself?—this seems also to be impossible. And, if the case of Joseph Pegg, whose ghastly appearance seems to have been so accurately pourtrayed by Mr. Bennet, was not small-pox, could the case of the young lady, who had been *satisfactorily* vaccinated fourteen years before, have been small-pox?

When Mr. Bennet's drawing of Joseph Pegg was shown to the mother of this young lady, she exclaimed, "Dear me! how like it is!" conceiving it to be the picture of her own daughter. Unfortunately, we are not informed by Dr. Bent (as, I think, we ought to have been), from what source this young person took the infection; nor whether true, that unquestionable small-pox prevailed at the time in her father's house, in the town of Derby, or in any of the neighbouring villages, to which she might happen to have paid a visit.

By *satisfactory* vaccination, I take for granted, was meant that the punctured arm became affected with the well-known vaccine pustule; that there was a crimson halo or burr spreading for several inches round that pustule; and that, on the seventh or eighth evening after the operation, she had some degree of fever: for I do not myself consider a patient as *constitutionally* vaccinated, unless this last symptom takes place; although I know my friend Mr. Ring, and many other eminent vaccinators, are perfectly satisfied with the first two symptoms, whether there is any fever or not.

Now, here is a string of cases *assumed* to be varioloid, without a single proof, or even (I will add) the slightest probability, of any variolous taint at all.

I have no doubt but the surgeons were right in pronouncing the eruptive disease of the children, who took the infection from Mary Miller, to be chicken-pox; and, although at so great a distance from the field of battle, I have no doubt or hesitation in pronouncing the case of the young lady to be a case of chicken-pox, and for this plain reason:—constitutional vaccination is an infallible preventive of true small-pox; this lady was, I have no doubt, constitutionally vaccinated; *ergo*, she could not take true small-pox; and it therefore follows, *à fortiori*, that her disease was not true small-pox.

Dr. Bent, who seems naturally to be friendly to vaccination, is staggered by these cases; but, if he will sit down and recollect himself a little, he will perceive that there is no ground for alarm. I am sorry to see such incorrectness of reasoning amongst medical men, whose logic should always approach to mathematical demonstration. But, I think, I can inform Dr. Bent how he may with certainty himself discover whether these cases were cases of chicken-pox or of small-pox.

Vaccination and variolation are the respective tests of each other: that is to say, inoculate a person, who has been constitutionally vaccinated, with small-pox matter, and he will not take true small-pox; inoculate a person, who has had the small-pox, with cow-pox matter, and he will not take the cow-pox. Let Mary Miller, then, Joseph Pegg, and the pupil at the hospital, (if they have not already been vaccinated or variolated,) be inoculated with vaccine or with small-pox matter; for, without one or the other, they are not secure against small-pox; and, if they take the small-pox, the disease they before had was chicken-pox; but, if they resist these inoculations, the disease may have been real small-pox. I conclude with observing, that, if the eruptive disease of these persons was not small-pox, it follows satisfactorily to my mind, as a necessary consequence, that none of the cases of eruptive disease, which prevailed in Derbyshire and Nottinghamshire, were cases of real small-pox; and I shall wait, therefore, with some degree of impatience for Dr. Bent's experiments.

P.S. In the years 1807 and 1808, there occurred in this town of Sherborne what I conceive to have been an eruptive disease; viz.—similar, if not identically the same, with that which has prevailed in Derbyshire and Nottinghamshire, which occasioned the same doubts and disputes which seem to have taken place there. Of this disease I published an account, in a letter to a friend in July 1818: this letter was spoken very well of in the Critical Review; but I do not recollect to have seen it noticed in any other Review or Journal, although I desired my bookseller to send it to your and all the Medical Journals; but I rather suspect he omitted to do so.*

This disease, like the Derbyshire eruption, affected indiscriminately those who had been vaccinated, and those who had neither been vaccinated nor variolated; but, at the time my book went to press, no case had come to my knowledge in which it attacked any person who had undergone the small-pox. Soon after my book was published, however, my friend, Mr. Gray, a surgeon of this town, desired me to

* This work was not transmitted to us.—Edit.

see a lad, who was nearly as much covered with papulous pustules as Joseph Pegg seems to have been, but he was not confined to his bed. This lad had been effectively inoculated for small-pox, seven years before, by the only medical anti-vaccinist in the town. This medical man was informed of the circumstance, and desired to see the lad; but he would not show, as the phrase is,—he would not look upon the patient. This occurrence led me to suspect (what indeed we had suspected before) that all the cases, except that of the lad mentioned in page 13 of my letter, were cases of chicken-pox only; but we chose to admit them all, for the sake of argument, to be cases of variola vaccinata, merely to guard against the nonsensical cavils of the anti-vaccinists. The reason why we did not see a case of this kind before, as well as the reason why we did not see more, may probably have been, that the proportion of persons vaccinated, as well as of those who had neither been vaccinated or inoculated for small-pox, might have been in the ratio of 100 to 1; scarcely any doubt having been before entertained that cow-pox was an infallible preventive of small-pox.

About the same time, some cases of real small-pox, after supposed real small-pox inoculation, occurred at Enmore Green, near to the neighbouring town of Shaftsbury, which occasioned me to make the following observations:—

How far the matter employed by Mr. Gray might, by possibility, without his knowledge, have been imperfect; how far we may, by possibility, have mistaken some other eruption for vaccinated small-pox; and how far the cases at Wareham, which are exactly in the same predicament, countenance or oppose such a supposition, I must leave to the conjectures of others: but the cases which occurred here were all so slight, as not materially, in my opinion, to affect the argument, whether small-pox or cow-pox inoculation should be preferred; but that mistakes and accidents are liable to happen with respect to small-pox, as well as to cow-pox inoculations, the following facts will sufficiently testify.

Mr. Adams, bookseller, at Shaftsbury, having mentioned to me that many persons at Enmore Green (a sort of suburb to Shaftsbury) had taken the real small-pox after supposed real small-pox inoculation, I desired him to collect the particulars, and send them to me; and in a few days he sent me the following letter:—

"Sir,

"Agreeably to your request, I have been at Enmore Green, where I found nine persons, [I learned afterwards that there were nearly twenty in the whole,] who had been inoculated for the small-pox by the late Mr. John White, about ten or eleven years ago, and were supposed to have had it *very fine*, and who have lately taken the small-pox in the natural way, and have had it *very severely*. I am, &c. &c.

"*Shaston; July* 16, 1808. T. Adams."

Now, the people at Enmore Green, as well as at Shaftsbury, firmly believed that these were cases of real small-pox after real small-pox, (of which I might have taken advantage, if I had been so disposed, as an argument against small-pox inoculation); but it requires very little penetration to discover that Mr. White had mistaken, as has frequently been done, a case of chicken-pox for a case of small-pox; had taken his matter from thence; and had communicated chicken-pox instead of small-pox to his patients.

These cases will, I think, convince Dr. Bent, that the great and immortal Sydenham, the respectable Dr. Morton, and Dr. Bent himself, were completely mistaken when they maintained small-pox and chicken-pox to be mere modifications of the same disease; since inoculation for small-pox does not prevent the infection of chicken-pox, nor does inoculation for chicken-pox (when mistakenly used) prevent the infection of small-pox;—and so much for the Derbyshire varioloid disease.

At the moment I was finishing the above, the Monthly Review for December 1818, and the Star paper of January 2nd, 1819, happened, by an extraordinary coincidence, to be lying on the table of my study; and, having finished what I intended to do, I took up the former, and, in the first article I perused, I met with the following paragraph:—

"The population of Ceylon has, of late, been greatly on the increase, [mark the reason assigned,] in consequence of the general introduction of vaccination."—*View of the Agricultural, Commercial, and Financial Interests of Ceylon;* by Anthony Bertolacci, Esq. 1818.

Having done with the Review, I took up the Star, and there I read the following advertisement:—

"An Account of the Small-Pox, as it appeared after Vaccination; by Alexander Monro, M. D. Including, among many cases, three which occurred in the author's own family."

Now, it has been shown above, on the authority of Mr. Bertolacci, that the population of Ceylon has been greatly increased by the general introduction of vaccination; and the different insurance offices will convince us that a similar increase has taken place in all countries into which vaccination has been generally introduced. Vaccination, therefore, is of great advantage to the population of the world at large.

3

The cases referred to by Professor Monro, admitting them for the moment to have been cases of real small pox after constitutional vaccination, must, no doubt, have been inconvenient (for it does not seem as if any of them had proved fatal) to Dr. Monro's family, as well as to the other families concerned: but of what sort of consequence are these cases, when compared to the welfare of the world at large?—they are but as a drop in the ocean. But, if these very cases should hereafter turn out (as is very probable) not to have been cases of real small-pox after constitutional vaccination, it is not merely nonsensical and ridiculous to trumpet them forth to the public, but it is, in my judgment, highly criminal to stir up the fears and resentments of the uninformed multitude against a practice which has done, is doing, and (if let alone) will continue to do, more in preserving the lives of the human species than all the lectures of all the professors, and all the practice of all the physicians, and surgeons, and apothecaries, in the universe, have done, are doing, or will ever do, as long as the world shall endure.

The beneficial or the prejudicial tendency of any grand measure or general practice, ought not to be estimated from its effect upon a particular individual, a particular family, or even upon many families, but from its general good or injurious effects upon large communities, or upon the great mass of mankind.

Notwithstanding that I am a decided advocate for vaccination, from a thorough conviction that nine-tenths, or rather that ninety-nine hundredths, of its reputed failures are cases which have arisen from imperfect vaccinations, or of cases taken for small-pox which were merely cases of chicken-pox; yet I am not amongst the number of those who would enforce the practice of vaccination, or prohibit the practice of small-pox inoculation, by Act of Parliament, because this would be contrary to Magna Charta, the Bill of Rights, and the other unalienable rights and privileges of Englishmen, who have the constitutional right of risking the lives of themselves, their children, and their neighbours, without let, hindrance, or molestation, whenever they think proper: but I would encourage vaccination by my advice, and by my example; and I would check and discourage inoculation for small-pox, not only by my advice and my example, but also by some salutary and constitutional restraints. If, then, it be lawful and constitutional to oblige an Englishman to take out a licence for killing game, it may perhaps, by analogy of law, be legal and constitutional to oblige an Englishman to take out a licence for killing his fellow-creatures. I would not, however, in this case recommend an annual licence, because this might be unequal in its pressure; but a licence, after the nature of a permit, upon a five-guinea stamp, for each individual offence; which, perhaps, might run somewhat in the following manner:—

"Permit Nicholas Noodle, of Fool's-alley, in the county of Middlesex, surgeon, to inoculate, with small-pox virus, Solomon, the son (or the only son, as the case might be,) of Solomon Softpate, esq. of Goatham Hall, in the same county."—Or,

"Permit Thomas Clutterbuck, of the city of Chester, surgeon and apothecary, to inoculate, with small-pox virus, Polly Higginbotham, of the same place, spinster."

"And if any physician, surgeon, apothecary, man or woman midwife, or other person pretending to skill in the art or science of physic, shall presume to inoculate any individual, without having first taken out such permit, he, she, or they, shall each and every of them forfeit and pay the sum of fifty pounds; one half to go to the informer, and the other moiety or half-part to the poor of the parish in which the offence shall be committed."

I have not had leisure to study, with due attention, the cases reported as *varioloid** after vaccination, by Dr. Thomson, of Edinburgh, and Mr. Hennen; but now that the cases which occurred at Ringwood, at Cambridge, at Wareham, at Sherborne, at Derby, at Nottingham, &c. &c. (which were all looked upon as varioloid,) have been proved, by competent judges, to have been cases of severe chicken-pox, I cannot help thinking, with all becoming deference to these gentlemen, that the disease which prevailed in the city and castle at Edinburgh were also cases of real but severe chicken-pox. Indeed, the cases at Lanark Mills, related by Dr. Thomson, appear to me to have been unquestionably cases of chicken-pox, and that for the following reasons:—as vaccination and variolation are universally admitted to be general preventives, at least, of future small-pox, the great proportion of those who had the disease after vaccination (like Mr. White's cases of small-pox after supposed small-pox, above mentioned,) proves too much; and, whatever proves too much, logically speaking, proves nothing.

Besides, there were four cases at these mills of this eruptive disease after *small-pox inoculation!* What, are the laws of nature reversed for the sole purpose of puzzling and perplexing us poor helpless mortals? Has small-pox, as

---

* This term was used by Dr. Thomson.—Edit.

well as cow-pox, lost its prophylactic power? The thing cannot be; there must be a mistake somewhere: either the vaccinations or the variolations must have been all ineffective, or the disease which occurred could not have been vario*lous* or vario*loid*.

"*Utrum horum mavis accipe.*"

Dr. Monro's book I have not seen, nor is it probable I ever shall see it. My mind has been long made up as to the unquestionable superior power of vaccination over even variolation, as a preventive of all danger from small-pox infection; and I have not the shadow of a doubt but the cases of Dr. Monro will speedily follow all the other supposed cases of small-pox after cow-pox to "the tomb of all the Capulets!"

"*Finis coronat opus.*"

"At Mr. Owen's mills," says Dr. Thomson, "through the obliging attention of Mr. Gibson, who has the medical charge there, I had an opportunity of seeing *one hundred and eighteen cases* [mark, one hundred and eighteen cases!] of young persons affected with this epidemic [*varioloid* disease after *vaccination*]. In its general appearance, the disease bore a very striking resemblance to that which I had occasion to see at Edinburgh; though, upon the whole, it appeared to me to have a character considerably milder. *Four only* of those affected with it had previously passed through *small-pox!* [so that Dr. Monro's children would not have been at all more secure against this disease, if they had been inoculated for *small*-pox instead of *cow*-pox]. In two of the cases after small-pox the disease was mild, but in the other two severe, [here is another proof that variolation is not a greater safeguard than vaccination]. Eighty-two had this disease after having passed through *cow-pox*. In a few of these, it might be said to be severe; but, in by far the greater number, it was extremely mild, and exhibited the most convincing and agreeable proofs of the efficacy of cow-pox in modifying small-pox."

So much, then, for the Scotch cases. I will add one fact of a different kind; but that one comprehending or representing many thousands of others, of the permanent effect of cow-pox in preventing small-pox.

About sixty years ago (which was long before vaccination was generally, if at all, known,) a servant of my father's, by name David Jervis, had been twice inoculated, in immediate succession, for the small-pox, without effect, by Mr. Clark, of Ansford in Somersetshire, who was at that time the most celebrated inoculator in the west of England, and who had not long before been in partnership with Mr. (afterwards the late Baron) Dimsdale, at Hertford. Mr. Clark was about to inoculate this man a third time; but of a sudden he said to him, "Pray, my friend, did you ever have the cow-pox?" The man replied, that he had it several years before. Mr. Clark, who was very quick in his manner, threw aside his lancet, and exclaimed, "G— d——, why didn't you tell me that before?" As if he had said, from his own immense experience, "I thought every fool knew that those who had once had the cow-pox could never take the small-pox."

I thought I had done; but, having delayed sending this paper longer than I intended, I have taken the opportunity of perusing with greater attention the cases related by Dr. Thomson and Mr. Hennen; and I must confess that they all confirm me still more in the opinion that I have delivered. The disease attacked indiscriminately those who had been vaccinated, those who had undergone the small-pox! and those who had neither undergone small-pox nor cow-pox. What is the natural inference? Surely not that the eruption was a varioloid eruption, after or produced by vaccination; for how could a non-eruptive disease produce an eruptive one?—not a varioloid disease, after or produced by small-pox inoculation; for how could the minimum or diminutive of small-pox produce effects which the maximum or augmentative of small-pox (that is to say, full malignant small-pox,) could not produce!—namely, infect those who had been before infected with real small-pox virus, and gone constitutionally through that disease? The thing is not only contrary to every analogy, but seems to be almost impossible.

What, then, is this eruptive disease?—Why, it is either a severe species of chicken-pox, or it is a disease, *sui generis*, long lost, but (as the miners speak) come again to day. And, after all, is it by any means clear that this disease has always, or even generally, prevailed after vaccination? I think not; unless that vaccination has been contemporaneously accompanied by the general prevalent small-pox. Vaccination, unlike small-pox, is practised day by day and hour by hour, as it were; because no one is afraid to vaccinate infants and persons of the most tender constitutions, nor of offending or injuring their neighbours by so doing: yet no varioloid disease ever follows these individual or isolated vaccinations; or, if they do, none such have come to my knowledge. But, after the general prevalence of small-pox, which is the ordinary cause of a general vaccination, the case is widely different. I have myself seen many instances of chicken-pox, as severe as that of Joseph Pegg, long before vaccination was generally known, or perhaps even thought of,—I mean so long ago as the year 1778; concerning which great disputes arose at the time, whether the complaint was small-pox or chicken-pox, insomuch that various practitioners inoculated with matter taken from one of the patients I allude to, under the persuasion that it was a case of actual small-pox; but to the misfortune of their patients; some of whom soon after took the real small-pox naturally, and some others were inoculated. These cases, in all, might have amounted to twenty or thirty, but only two or three were of the severe kind above spoken of; and all of these I now allude to occurred in the year next after the general prevalence of small-pox, but whether they had any connexion with real small-pox as their cause or their modification, I confess myself unable to unravel; but the eruption was so much like small-pox, that nothing but the greater milkiness of the pustules, their want of the pyramidal form, of the crimson base, their more speedy drying-off, and the absence of the variolous fœtor, enabled me to pronounce with confidence that the disease was not small-pox: and, at all events, the eruptive disease which occurred at Edinburgh, at Derby, perhaps at Montpellier, and in many other places abroad and at home, may as justly be called a varioloid disease after small-pox as a varioloid disease after cow-pox; so that the reputation of small-pox inoculation, as a preventive of small-pox, obtains no additional advantage over vaccination by the occurrence of these cases.

"*Parturiunt montes et mus nascitur.*"

The reason why so many more instances of this eruptive disease occurred after vaccination than after variolation, and which seems to be the sole foundation of the present controversy, I take (as I have hinted before) to be this:—the practice of vaccination, until this ill-founded—and I hope, for the benefit and honour of human nature, temporary—slur upon its character took place, had become almost universal, and was opposed only by a few straggling individuals, who might be called *the wild men of the woods!* and Dr. Thomson's cases (which, by the bye, considering the population of Edinburgh, 122,954 persons, are very few—only 72,) do, in a most extraordinary manner, confirm this supposition: for, out of the 72 eruptive cases, 37 of the patients had never been vaccinated or variolated, 27 had passed through cow-pox. Now, 27 is rather less than one-third of 72, the whole number; and 8, the number who had passed through small-pox, is rather less than one-third of 27, the number who had passed through cow-pox: the proportions, therefore, between the vaccine failures and the variolous failures (supposing, for the moment, that they were failures at all,) are very exactly preserved; and the prophylactic power of vaccination is, according to these cases, to the full as great as the prophylactic power of variolation, which was the question to be investigated. But it is still more remarkable how nearly the aggregate number of those who had been vaccinated and variolated corresponds with those who had neither been vaccinated nor variolated: 35, the number so guarded, being, all but *half a patient*, the moiety of 72, the whole number affected. And what does this go to prove?—Why, to my mind, it goes to prove that there was *no small-pox infection to prevent*, and that every one of these cases, both at home and abroad, were no other than cases of *chicken-pox*.

If, after all that has been said by others, as well as by myself, there should still be any medical man blind and foolish enough to prefer, and any parents weak enough to permit, variolation to be practised on their children, I can only say

"*Si populus vult decipi decipiatur.*"

But I will declare it as my settled opinion, that, if all parents would have their children vaccinated in their first year, then that horrible pestilence, the small-pox, would in two short years be entirely driven from our land:—" a consummation devoutly to be wished."

More new light still.—During the time I was writing the above, a very respectable and well-educated female came to consult me: she has for thirty-five years past kept a day-school in this town, and has had during all that time, upon an average, more than forty scholars, most of them under ten years of age; so that, multiplying 35 by 40, she has had about 1400 scholars under her tuition. She is one of those persons who are generally called motherly women, and she has acquired the knack, either from love or fear, according to their respective dispositions, of commanding the obedience of the children; and therefore, when any of her little pupils are unwell, her attendance is almost always requested, either to give her advice or to prevail on the children to take their medicines. It occurred to me to ask this person whether she ever recollected to have seen a disease called the swine-pox, as a disease distinguishable from chicken-pox?—she said, she did not recollect to have seen any such disease; " but," says she, " I have known many instances of children having the chicken-pox twice; and at one of those times the disease has been always very severe, and so much like small-pox, that it was very difficult to distinguish it from small-pox." Upon my asking her if she could recollect whether the severe disease or the mild disease regularly occurred first? she said, she could not recollect any difference; but she was inclined to think that one occurred first as frequently as the other, and that it was only a few, amongst a considerable number of mild cases, which put on this severe form.

I am really sorry to bother the public with so much of the *crambe recocta* of small-pox and cow-pox inoculation; but I confess that I am extremely anxious, by every fair and legitimate argument, to establish a practice which appears to me so highly beneficial and important to mankind.

*Sherborne; Feb.* 1819.

